# Equine herpesvirus type 1 infection induces procoagulant activity in equine monocytes

**DOI:** 10.1186/1297-9716-44-16

**Published:** 2013-03-11

**Authors:** Wee Ming Yeo, Nikolaus Osterrieder, Tracy Stokol

**Affiliations:** 1 Department of Population Medicine and Diagnostic Sciences, College of Veterinary Medicine, Cornell University, Ithaca, NY, USA; 2 Institut für Virologie, Freie Universität Berlin, Philippstraße 13, Berlin, 10115, Germany

## Abstract

The alphaherpesvirus, equine herpesvirus type 1 (EHV-1), is a highly prevalent cause of equine infectious abortion and encephalomyelopathy. These syndromes have been attributed to ischemic necrosis from thrombosis in placental and neural vessels, although the mechanisms underlying thrombosis are unknown. After inhalation, EHV-1 establishes a peripheral blood mononuclear cell-associated viremia, with monocytes being a target of infection. Monocytes are also the main source of tissue factor (TF) in diseased states. Since TF is the primary activator of coagulation, increased monocyte TF expression could be involved in EHV-1-associated thrombosis. We hypothesized that EHV-1 infection would induce TF-dependent procoagulant activity in equine monocytes. Monocyte-enriched fractions of blood were infected with abortigenic (RacL11, NY03) and neuropathogenic (Ab4) EHV-1 strains. All strains induced procoagulant activity, to variable degrees, within 1 to 4 h, with maximal activity at 24 h, after infection. Virus-induced procoagulant activity was similar to that seen with lipopolysaccharide, a known stimulant of TF-mediated procoagulant responses. Virus-induced procoagulant activity was factor VIIa-dependent and temporally associated with TF gene transcription, implicating TF as the main driver of the activity. Procoagulant activity was mildly decreased (30-40%) when virus was inactivated by ultraviolet light or when infected cells were treated with aphidicolin, a virus DNA polymerase inhibitor, suggesting early events of virus infection (attachment, entry or intracellular trafficking) are the primary stimulus of procoagulant activity. Our results indicate that EHV-1 rapidly stimulates procoagulant activity in equine monocytes in vitro. The EHV-1-induced procoagulant activity in monocytes may contribute to clinical thrombosis in horses with EHV-1 infection.

## Introduction

Equine herpesvirus type 1 (EHV-1) is a member of the genus Varicellovirus within the Alphaherpesvirinae subfamily and has a double stranded DNA genome. It is highly prevalent and pathogenic in horses, causing large-scale outbreaks of respiratory disease, abortion storms and encephalomyelopathy [1]. Horses are exposed to EHV-1 through aerosolization [1,2]. After primary replication in the respiratory epithelium, the virus disseminates systemically through a peripheral blood mononuclear cell (PBMC)-associated viremia [3-7]. The EHV-1-associated disease syndromes of abortion and encephalomyelopathy have been attributed to ischemic injury from thrombosis in vessels of the placenta and the central nervous system, mostly in the spinal cord, respectively [8-10].

Infectious agents, including bacteria and viruses, can activate the hemostatic system, resulting in a hypercoagulable state that can manifest as thrombosis or disseminated intravascular coagulation [11-13]. Experimental models have revealed that upregulation of tissue factor (TF) expression on monocytes is the main trigger for hypercoagulability in bacterial sepsis [14,15], however the mechanisms underlying hemostasis activation with virus infection are less well understood. In vitro studies have shown that viruses can upregulate TF in infected endothelial cells [16-18]. However, monocytes are now considered the main source of TF in inflammatory states, including that due to bacterial sepsis [14,19]. Far less is known of the role of monocytes in virus-induced hypercoagulability. Four bovine respiratory viruses upregulated TF in alveolar macrophages in vitro [20] while cytomegalovirus (a betaherpesvirus) and influenza virus induced TF-dependent procoagulant activity in human monocytes in vitro [21]. In vivo studies have associated TF expression on monocytes with abnormalities in hemostasis in several virus infections [22-24].

The mechanisms of thrombosis in EHV-1 infection have not been established, although endothelial injury certainly may be involved [3,9,10]. However, blood monocytes are susceptible to EHV-1 infection in vitro and in vivo [2,5,25-27] and may serve as the primary virus transporters to endothelial cells at sites distant from the respiratory tract [2,28,29]. Since monocytes are also the source of induced TF expression in disease states, we hypothesized that EHV-1 infection would induce procoagulant activity in equine peripheral blood monocytes in a TF-dependent manner. Monocyte-enriched fractions from equine peripheral blood were infected with abortigenic (RacL11, NY03) and neuropathogenic (Ab4) strains of EHV-1. At various times after infection with live or inactivated virus, monocyte procoagulant activity was measured with a two-stage amidolytic activity assay, based on the generation of activated factor X (FXa). TF mRNA was also quantified at different times after infection and flow cytometric analysis was performed to verify infection of monocytes by EHV-1 and determine if EHV-1 induced cell death in infected monocytes.

## Materials and methods

### Virus amplification, concentration and titration

The following EHV-1 strains were used: RacL11_D752_, Ab4_D752_, NY03_N752_, Ab4_D752_ expressing green fluorescent protein (Ab4_D752_-GFP), RacL11_N752_-GFP and Ab4_N752_-GFP. The RacL11 strain was isolated from an aborted fetus in the 1950s [30], whereas Ab4 was isolated from a quadriplegic mare that had previously aborted during an EHV-1 outbreak in 1980 [9]. NY03 is an isolate from an aborted fetus in the state of New York in 2003 [31]. Wild-type RacL11 and Ab4 strains contain a single G to A polymorphism in the catalytic subunit of the EHV-1 DNA polymerase at position 2554, which results in a single amino acid substitution of aspartic acid (D) for asparagine (N) at position 752 (D_752_) that was shown to be associated with neurological disease [25,27,32,33]. The NY03 strain has a G nucleotide at this position (N_752_). The mutant viruses, RacL11_N752_-GFP and Ab4_N752_-GFP, were generated by substituting A for G, as previously described [25]. GFP-expressing strains were produced by substituting the GFP gene for the virus gene 71, which encodes for the nonessential glycoprotein gp2, as previously described [34]. A clinical equine herpesvirus type 4 (EHV-4) field isolate was kindly provided by Dr Dubovi (Cornell University). All EHV-1 strains were propagated in rabbit kidney cells (RK13), which were cultured in Dulbecco’s Modification of Earl’s Medium (Mediatech, Inc.) supplemented with L-glutamine, glucose, sodium pyruvate and 10% fetal bovine serum (Atlanta Biologicals, Inc.) and grown in 75 cm^2^ flasks (Corning Inc.) at 37°C under 5% carbon dioxide. One-day-old confluent monolayers of RK13 cells were infected with the respective virus strains at a multiplicity of infection (MOI) of 0.1 until a cytopathic effect of approximately 90% was achieved. The flasks were then subjected to two freeze-thaw cycles. The respective culture media were centrifuged at 1800 × *g* for 15 min at 4°C, aliquoted, and subjected to ultracentrifugation at 175 000 × *g* for 1 h at 4°C. The supernatant was removed (and stored for later use as a negative control) and the pellet was reconstituted with 1 mL sterile phosphate-buffered saline (PBS, pH 7.4) and stored at -20°C. The virus titers of the respective concentrates were determined using a standard plaque assay.

### Virus infection of monocyte-enriched and monocyte-depleted fractions of peripheral blood mononuclear cells

Peripheral blood was collected with minimal restraint on multiple different occasions from the jugular vein of 14 non-pregnant Thoroughbred mares ranging in age from 3 to 20 years, using a blood collection set (40 inch, MWI Veterinary Supply). Blood was collected directly into a 250 mL evacuated container (Baxter Healthcare Corporation), containing heparin (10 units/mL, Sigma-Aldrich). All animal procedures were approved by the Cornell University Institutional Animal Care and Use Committee and were in accordance with the guidelines established by the National Institutes of Health. Leukocyte-rich plasma was obtained by gravity sedimentation of erythrocytes for 20 min at room temperature. The plasma was then centrifuged at 288 × *g* for 10 min at 20°C. The cell pellet was resuspended with PBS (pH 7.2), then layered onto Histopaque 1.077 (GE Healthcare) at a ratio of 2:1 cell suspension to Histopaque and centrifuged at 468 × *g* for 15 min at 20°C. The PMBC layer was removed, washed twice in PBS, and then was resuspended in MACS buffer (MACS, Miltenyi Biotec). The cell suspension was incubated with a murine monoclonal anti-equine CD14 antibody (clone 105, 20 μg/mL) [35] for 15 min at 4°C. Antibody-bound cells were positively selected by incubation with a secondary rat anti-mouse antibody conjugated to metal beads (Miltenyl Biotec, 1:100) for 15 min at 4°C followed by passage through a magnetic column as recommended by the manufacturer. The sorted (monocyte-enriched) and flow through (monocyte-depleted) fractions were washed in PBS and resuspended in RPMI medium containing 10% autologous plasma that had been rendered free of cell-derived microparticles by two steps of high-speed centrifugation at 21 130 × *g* for 30 min at 4°C. The percentage of monocytes in each fraction was determined by performing differential cell counts on 200 leukocytes in Wright’s-stained cytospin smears prepared from each fraction immediately after sorting. The cells were seeded at a density of 5 × 10^5^ cells/well in 96-well tissue culture plates and immediately infected with the various EHV-1 strains at an MOI of 1 or 5 for 1 h at 37°C. The infected cells were then washed twice with warmed PBS to remove non-infectious virus particles and fresh media was added. Vehicle (PBS) and lipopolysaccharide (LPS, 10 ng/mL, Sigma-Aldrich) served as negative and positive [36] controls, respectively. Infected and control (uninfected) cells from the same horse were incubated simultaneously for various times (1, 2, 4 or 24 h) in a humidified chamber at 37°C with 5% carbon dioxide. In some experiments, viruses were inactivated by exposure to ultraviolet (UV) light for 20 min before infection or infected cells were treated with the virus DNA polymerase inhibitor, aphidicolin (10 μM, Sigma-Aldrich), which was added after 1 h of virus adsorption.

### Measurement of procoagulant activity on monocyte surfaces

Infected and control cells (5 × 10^5^) were washed twice with HEPES buffer (10 mM HEPES, 137 mM sodium chloride, 5 mM calcium chloride, 4 mM potassium chloride, 10 mM glucose, 0.5% bovine serum albumin, pH 7.4), then procoagulant activity was measured using a standard two-step amidolytic assay based on FXa generation. In brief, recombinant human activated factor VII (FVIIa, final concentration, 1 nM, Hematologic Technologies) and factor X (FX, final concentration, 75 nM, Hematologic Technologies) diluted in HEPES buffer were added to the cells for 15 min at 37°C. Chromogenic substrate (Spectrozyme-FXa, final concentration, 167 μM, American Diagnostica) was then added and the ensuing color change was measured after 10 minutes at an optical density of 405 nm at 37°C with a plate spectrophotometer (Synergy 2, Biotek, Winooski, VT, USA). Optical density results were converted to the amount of FXa generated (nM) based on a standard curve created from serial dilutions of human recombinant FXa (American Diagnostica) that were incubated with the substrate for 10 min. The standard curve was linear between FXa concentrations of 4.69 and 0.29 nM, the latter yielding optical densities more than twice baseline values (infected or control cells with no exogenous FX or chromogenic substrate). For data analysis, values above and below the linearity of the standard curve were standardized to 4.69 and 0 nM FXa, respectively. Human recombinant TF (Innovin, Dade-Behring) with added reagents was included as a positive assay control.

### Quantitative real-time PCR for TF mRNA

Total RNA was isolated with the RNeasy mini kit (Qiagen), using RNase-Free DNase, according to the manufacturer’s protocol. Complementary DNA (cDNA) was synthesized using Superscript III first strand reverse transcriptase kit (Life Technologies) according to the manufacturer’s protocol. Resulting cDNA concentrations were measured at 260 nm with a nanodrop spectrophotometer (NanoVue, GE Healthcare). Gene-specific and housekeeping gene (equine β2-microglobulin) primers were designed using the NCBI primer blast program and the final sequences were verified against the available mammalian genome database (NCBI/BLAST) to avoid non-specific homology (Table 1). Quantitative PCR was performed on 200 ng cDNA samples using PowerSybr green in a MicroAmp Fast Optical 96-well reaction plate (Applied Biosystems) according to the manufacturer’s protocol. Samples were run in duplicate or triplicate on an ABI StepOnePlus Real Time PCR system in standard mode, with 10 min of denaturation at 95°C, followed by 40 cycles of 15 s at 95°C and 60 s at 60°C and 60 s at 72°C. Melting curve analysis was used to confirm the presence of a single PCR product. The data were analyzed with the ΔΔCT method [37], with results being normalized to the housekeeping gene and expressed as the log_2_ fold difference in target quantity between the test sample and vehicle-treated control (set to 0).

**Table 1:** Primers used for measurement of equine TF mRNA by quantitative real-time PCR

**Gene**	**Length (bp)**	**Amplicon size**	**Sequence (5’ – 3’)**
Equus caballus TF forward	23	243	5' - GTGGCTAGAGCCGCAGGGACTAG
Equus caballus TF reverse	21	243	5' - AGGAAGAGACCCGCGCCATGT
Equus caballus β2-microglobulin forward	22	209	5’ - TGTCTCTGGGTTCCATCCGCCT
Equus caballus β2-microglobulin reverse	25	209	5’ - CGGACCCACTTAACTATCAGGGGGT

### Flow cytometric analysis of virus infectivity of monocytes

Monocyte-enriched and -depleted cultures were infected with RacL11_N752_-GFP and Ab4_D752_-GFP at an MOI of 1 for 24 h, with vehicle (PBS) and non-GFP-expressing virus as controls. The samples were then labeled with an Alexa647 (A647)–conjugated murine anti–equine CD14 antibody (clone 105) or an A647-conjugated isotype control for 15 min at 4°C and washed three times with PBS. Then 15 000 cells/sample were analyzed on a FACSCaliburTM flow cytometer (Becton Dickinson) using the following settings: forward scatter (FSC): E00 voltage, 1.14 amp gain, linear mode, threshold 180; side scatter (SSC): 434 voltage, 1.00 amp gain, linear mode; and fluorescence on log mode. The percentage of cells that were positive for A647-CD14 or GFP was measured on quadrant dotplots.

### Flow cytometric assessment of cell death

To determine if EHV-1 induced cell death, we infected cells for 4 or 24 h with all EHV-1 strains at an MOI of 1 and 5 and then measured changes in mitochondrial transmembrane potential (ΔΨm) by staining with a fluorescent cationic dye and monitoring changes in fluorescence with flow cytometry, according to the manufacturer’s protocol (DePsipherTM kit, Trevigen Inc.). For analysis, monocytes were gated based on their FSC and SSC characteristics. In healthy cells with a high negative ΔΨm, the lipophilic cationic dye accumulates in the mitochondrial matrix as a multimer and fluoresces bright red (FL2 channel). Red fluorescence is lost when the cells have altered mitochondrial membrane permeability, an early marker of apoptosis [38]. Since changes in ΔΨm are potentially reversible [39], we also performed a live-dead assay by staining for Annexin V and measuring propidium iodide uptake (TACS® Annexin V-FITC kit, Trevigen Inc), according to the manufacturer’s protocol. This was done on monocyte-enriched fractions infected with RacL11 at an MOI of 1 or 5 for 4 or 24 h. Cells treated with LPS (10 ng/mL or 10 μg/mL) and sodium azide (25 mM, Sigma-Aldrich) served as positive treatment and cell death controls, respectively, whereas vehicle- (PBS) treated cells were used as a negative control for all cell death experiments.

### Statistical analysis

Data were parametric and results are expressed as mean ± SD values. Comparison between multiple means was performed with a 1-way ANOVA followed by a Tukey’s Multiple Comparison Test. Comparison of two means was performed with a paired t-test (Prism 5.0 for Mac OS, version 5.0c, GraphPad Software Inc). Significance was set at *p* < 0.05.

## Results

### EHV-1 infection of monocytes and induction of procoagulant activity

**Figure 1:**
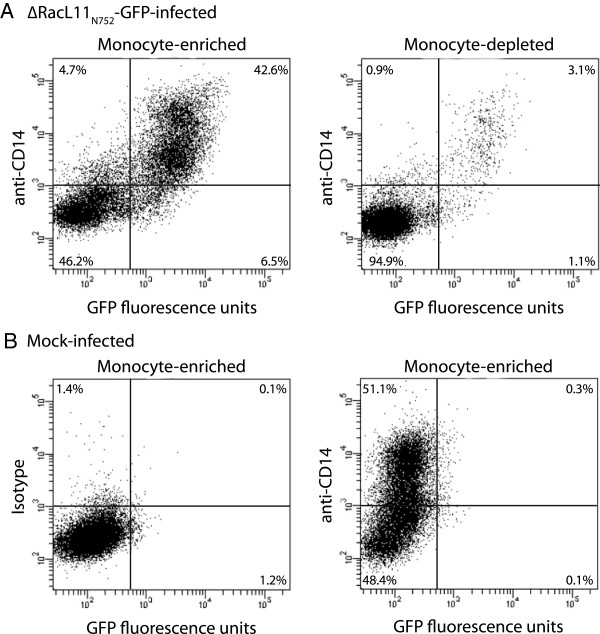
**EHV-1 preferentially infects equine monocytes in peripheral blood mononuclear cells.** Monocytes were enriched from equine PBMC using an anti-equine CD14 antibody and immunomagnetic beads. Monocyte-enriched (**A**, left panel) and -depleted (**A**, right panel) fractions of PBMC (5 × 10^5^ cells) were infected for 24 h with RacL11_N752_-GFP (MOI of 1), then labeled with an A647-conjugated anti-CD14 antibody, after which 15 000 cells were analyzed for EHV-1 infection (GFP-positivity) by flow cytometry. Mock-infected monocyte-enriched fractions labeled with an A647-isotype antibody (**B**, left panel) or A647-conjugated anti-CD14 antibody (**B**, right panel) served as negative controls, with the isotype control being used to define the quadrants. Numbers in each quadrant represent percentage of total events. The majority of GFP-positive cells (upper and lower right quadrants) in EHV-1-infected monocyte-enriched and -depleted fractions are CD14-positive monocytes (upper right quadrant).

To verify that monocytes were infected with EHV-1 in vitro, flow cytometric analysis with the GFP-expressing variants RacL11_N752_ and Ab4_D752_ was done. A monocyte purity of 81 ± 7% (range 65-88%, *n* = 10) was obtained in the monocyte-enriched fraction with the anti-equine CD14-based immunopurification technique, as assessed by differential leukocyte counts on cytospin preparations of the fraction. Flow cytometric analysis was performed 24 h after infection of monocyte-enriched and -depleted fractions to allow time for virus infection and replication. Monocytes were the predominant cell type infected with the RacL11 strain (43.9 ± 2.2%) in both fractions (Figure 1). Similar observations were made when the Ab4 strain was used (data not shown), but infection rates were slightly lower with this strain (38.4 ± 2.8%).

**Figure 2:**
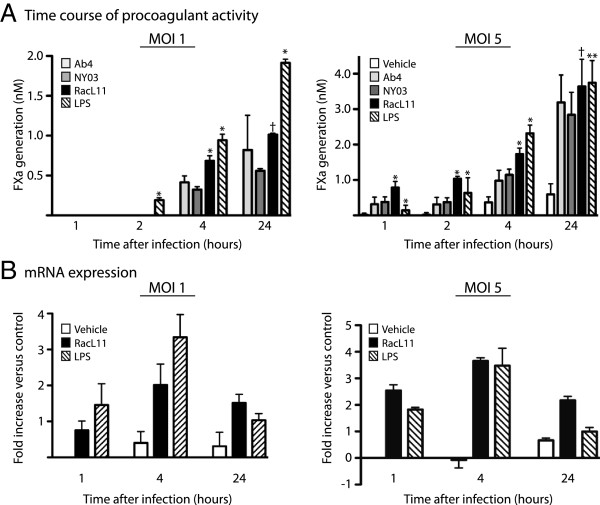
**Time course of procoagulant activity and mRNA expression after EHV-1 infection of equine monocytes. A**: Monocyte-enriched fractions of equine PBMC (5 × 10^5^ cells) were infected with the RacL11, Ab4 or NY03 strains of EHV-1 at an MOI of 1 (left panel, *n* = 4) or 5 (right panel, *n* = 10) or treated with LPS (10 ng/mL) positive or vehicle (PBS) negative controls. Procoagulant activity was measured at 1, 2, 4 and 24 h after infection. FXa generation was below the limit of detection at all time points for the vehicle control and at 1 and 2 h after infection for all viruses at an MOI of 1 and are subsequently not shown in the left panel. The mean procoagulant activity of cells infected with all viruses or treated with LPS is significantly different than the vehicle control (*p* < 0.05) at all time points, with the following exceptions: 1 or 2 h after infection with all virus strains at an MOI of 1 (left panel) and 1 h after treatment with LPS (both panels). Note, results depicted in the two panels are derived from different horses and experiments. * *p* < 0.05 versus other virus strains (for RacL11) or all virus strains (for LPS) at the same time point. ** *p* < 0.05 versus Ab4 or NY03 only. † *p* < 0.05 versus NY03 only. **B**: Time course of quantitative real-time PCR analysis for TF mRNA in monocyte-enriched PBMC fractions infected with RacL11 at an MOI of 1 (left panel) or 5 (right panel) or treated with LPS (10 ng/mL) and vehicle (PBS) controls. The data is expressed as the mean ± SD log_2_ fold increase in TF mRNA expression relative to the vehicle-treated control at 1 h (standardized to 0) after normalization to the housekeeping gene, β2-microglobulin (*n* = 3–6).

Monocyte-enriched and -depleted fractions were then infected with the RacL11 strain of EHV-1 at an MOI of 5 for 4 h, using LPS (10 ng/mL) as a positive control. This time point was initially chosen because it is close to the peak procoagulant activity previously reported for LPS-stimulated equine monocytes [36]. Both RacL11 infection and LPS treatment caused a significant increase in procoagulant activity in the monocyte-enriched fraction compared to the monocyte-depleted fraction and vehicle-treated controls. The increase in procoagulant activity was significantly higher after LPS stimulation than after RacL11 infection (Table 2). The procoagulant activity of both negative controls and RacL11-infected or LPS-treated monocyte-depleted fractions were below the assay detection limit. Two other EHV-1 strains, Ab4 and NY03, also induced procoagulant activity in equine monocyte-enriched fractions after 4 h of infection, although the response was highest with the RacL11 strain (Figure 2A). Because inhibitory antibodies against equine TF are unavailable, controls lacking exogenous FVIIa were used as a surrogate marker of TF involvement in the induced procoagulant activity. The increase in procoagulant activity after infection with all EHV-1 strains (MOI of 1 or 5) and LPS stimulation was abolished in the absence of exogenous FVIIa (Table 3, only RacL11 is shown at an MOI of 1) supporting TF-triggered, FVIIa-dependent generation of FXa. The induced procoagulant activity was not due to LPS contamination of purified virus because virus-free supernatants obtained after ultracentrifugation did not stimulate any procoagulant activity in the monocyte-enriched fractions (data not shown). Other herpesviruses can acquire TF from the membranes of the host cells they are propagated in [40]. All three purified virus alone (tested at concentrations similar to an MOI of 5) expressed small amounts of TF that were usually below the limit of detection of the assay (0.19 to 0.39 nM FXa), but higher than the negative controls (virus preparations with no added FX or substrate). The amount of FXa generated by the virus alone was far lower than that induced by virus infection or LPS stimulation of cells. This data suggests that EHV-1, similar to other herpesviruses, do acquire TF from the propagating rabbit kidney cellular membranes, but the small amount of procoagulant activity on the purified virus is insufficient to account for the observed activity in infected equine monocytes.

**Table 2:** EHV-1 induces procoagulant activity primarily in the monocyte-enriched fraction of equine peripheral blood mononuclear cells

**Treatment**	**Vehicle**	**RacL11**	**LPS**
Monocyte-enriched*	0	2.36 ± 0.05**	2.78 ± 0.07**
Monocyte-depleted	0	0	0

Monocytes were purified using a murine anti-equine CD14 antibody and procoagulant activity was measured in monocyte-enriched and -depleted PBMC fractions with a two-stage amidolytic assay 4 h after infection with RacL11 (MOI of 5), using LPS (10 ng/mL) positive and vehicle (PBS) negative controls. Data represents the mean ± SD of FXa generation (nM) from 3 horses.

* *p *< 0.05 comparing all treatments for monocyte-enriched cells.

** *p *< 0.05 versus the monocyte-depleted fraction.

**Table 3:** EHV-1- and LPS-induced procoagulant activity is FVIIa-dependent

**Treatment**	**Vehicle**	**RacL11**	**LPS**
FVIIa	0	1.97 ± 0.27*	2.89 ± 0.37*
No FVIIa	0	0	0

Procoagulant activity (mean ± SD FXa generation in nM) was measured with and without exogenous FVIIa in monocyte-enriched PBMC fractions 4 h after infection with RacL11 (MOI of 1) or treatment with LPS (10 ng/mL) or vehicle (PBS) (*n* = 3).

* *p* < 0.05 versus no FVIIa.

### Time course of EHV-1 induction of procoagulant activity and TF mRNA expression in equine monocytes

Procoagulant activity was measured in monocyte-enriched fractions at 1, 2, 4 and 24 h after infection with all 3 virus strains at an MOI of 1 or 5 in different experiments. At an MOI of 1, procoagulant activity was evident at 4 h after EHV-1 infection. At this MOI, procoagulant activity at earlier time points of EHV-1 infection and in all vehicle treated controls was below the assay detection limit (Figure 2A, left panel). In contrast, at an MOI of 5, procoagulant activity was observed as soon as 1 h after virus infection and was significantly higher with all three virus strains compared to vehicle negative and LPS positive controls at this time point (Figure 2A, right panel). Furthermore, the induced procoagulant activity was higher at an MOI of 5 compared to an MOI of 1 at all time points with all virus strains (Figure 2A, left versus right panel). Procoagulant activity was maximal 24 h after infection at both MOI or LPS stimulation. RacL11 consistently induced the highest procoagulant activity of all virus strains (Figure 2A). Basal procoagulant activity and the degree of change in procoagulant activity varied between individual horses and experiments. Similar variability in basal and stimulated procoagulant activity is a recognized phenomenon with human monocytes [41].

To determine if the induction of procoagulant activity due to EHV-1 was associated with TF gene transcription, quantitative real-time PCR was performed at 1, 4 and 24 h after EHV-1 infection at an MOI of 1 and 5 or LPS stimulation. An increase in TF mRNA expression occurred within 1 h of infection with RacL11 at both MOI, which paralleled the TF mRNA response to LPS (Figure 2B). Similar to changes in procoagulant activity, there was a greater relative increase in TF mRNA expression at the higher MOI of 5 at all measured time points (Figure 2B, left versus right panel). The upregulated mRNA expression peaked at 4 h (preceding the peak procoagulant activity), then began to return to baseline levels at 24 h after infection or stimulation (Figure 2B). Similar results were observed with the Ab4 and NY03 EHV-1 strains at an MOI of 5 (data not shown).

### Effect of the D_752_ polymorphism in the virus DNA polymerase on EHV-1-induced procoagulant activity

The RacL11 -and Ab4 EHV-1 strains both contain the D_752_ polymorphism, which has been associated with neurovirulence [25,27,32,33], whereas the NY03 strain has the N_752_ polymorphism in the virus DNA polymerase. The observation that all 3 EHV-1 strains induced procoagulant activity in equine monocytes, albeit to different degrees (Figure 2), argues against a major role for this known pathogenic polymorphism in the induction of procoagulant activity. To more directly determine the effect of this polymorphism on procoagulant activity, monocyte-enriched fractions were infected with wild type EHV-1 RacL11_D752_ and Ab4_D752_ and isogenic mutants expressing N_752_ (RacL11_N752_ and Ab4_N752_). Both parental and mutant viruses induced procoagulant activity in monocytes, although the degree of induction was significantly lower with the N_752_ mutant of RacL11 only (Table 4).

**Table 4:** **Effect of the D**_**752 **_**polymorphism in the virus DNA polymerase on EHV-1-induced procoagulant activity**

**Infection**	**FXa generation (nM)**
None (vehicle)	0
RacL11_D752_	2.24 ± 0.18*
RacL11_N752_	1.71 ± 0.02
Ab4_D752_	1.85 ± 0.10
Ab4_N752_	1.78 ± 0.08

Monocyte-enriched PBMC fractions were infected with wild type RacL11_D752_ and Ab4_D752 _strains and engineered mutant strains (RacL11_N752_, Ab4_N752_) at an MOI of 5. Procoagulant activity was measured at 4 h after infection or vehicle (PBS) treatment (*n *= 4).

* *p* < 0.05 versus mutant strain. All EHV-1 infected cells had significantly higher mean procoagulant activity than vehicle-treated cells.

### Effect of virus inactivation and EHV-4 infection on monocyte procoagulant activity

**Figure 3:**
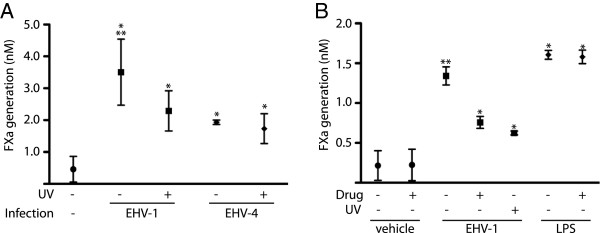
**Inactivation of virus with UV light or aphidicolin treatment decreases EHV-1-induced monocyte procoagulant activity. A**: UV light inactivation decreases procoagulant activity induced with the RacL11 strain of EHV-1, whereas it has no effect on procoagulant activity induced by EHV-4 (*n *= 3). * *p *< 0.05 versus vehicle negative control. ** *p *< 0.05 versus EHV-4-infected cells (active or inactivated). **B**: Aphidicolin (10 μM) decreases the procoagulant activity induced by the RacL11 strain of EHV-1, but has no effect on basal procoagulant activity or that induced by LPS (10 ng/mL) (*n *= 3). The degree of inhibition of procoagulant activity with UV light inactivation and aphidicolin are similar. Note, results depicted in Figure 3A and B are derived from different horses. * *p *< 0.05 versus untreated or drug-treated vehicle (PBS) control. ** *p *< 0.05 versus UV light-exposed or aphidicolin-treated virus.

To determine if procoagulant activity was induced by early or immediate early (pre-replication) phases of virus infection (attachment, entry or intracellular trafficking) in monocytes, RacL11 was inactivated by exposure to UV light; UV-exposed virus can presumably attach, enter and traffic to the nucleus of cells but is incapable of replication, which was confirmed through virus plaque assays (data not shown). Procoagulant activity was significantly decreased by 29 ± 5% at 4 h of infection when UV-inactivated RacL11 was used at an MOI of 5 (Figure 3A). Similarly, mild decreases in procoagulant activity were observed when monocytes were infected with UV-inactivated Ab4 (31 ± 1% decrease) and NY03 (35 ± 1% decrease). The data suggested that procoagulant activity is mainly induced by events in the early phases of virus infection, but that synthesis of late virus proteins with virus replication contributes to and maximizes the activity. To test this hypothesis, we treated monocytes with the virus DNA polymerase inhibitor, aphidicolin (10 μM) [42], during RacL11 infection. Treatment with aphidicolin decreased the procoagulant activity induced by RacL11 infection to a similar extent as UV inactivation (aphidicolin: 29 ± 3%; UV inactivation: 35 ± 5%) (Figure 3B). Similar decreases in procoagulant activity were observed in monocytes treated with aphidicolin and infected with Ab4 (25 ± 16% decrease) and NY03 (40 ± 4% decrease). However, the drug had no effect on basal or LPS-stimulated procoagulant activity (Figure 3B). The data indicated that synthesis of late virus proteins contributes to the observed procoagulant activity in EHV-1-infected monocytes. Since most of the procoagulant activity was induced by pre-replication stages of virus infection, we sought to determine if the procoagulant response was specific to EHV-1 or could be induced by a related herpesvirus. We thus infected monocytes with EHV-4 at an MOI of 5. We chose EHV-4 because this virus does infect PBMC [43] and mononuclear cells in nasal explants [44] in vitro but is not associated with thrombosis in vivo. We found that EHV-4 did induce procoagulant activity in equine monocytes and that this activity was also dependent on exogenous FVIIa (data not shown), however the response was significantly lower than that observed with EHV-1 and was similar to that seen with UV-inactivated EHV-1. Furthermore, unlike EHV-1, the EHV-4-induced procoagulant response was unaffected by UV inactivation (Figure 3A) or aphidicolin treatment (data not shown). We concluded from the data that only the early phases of EHV-4 infection induce procoagulant activity and that EHV-4 replication does not contribute to the phenomenon.

### Monocyte cell death after EHV-1 infection

**Figure 4:**
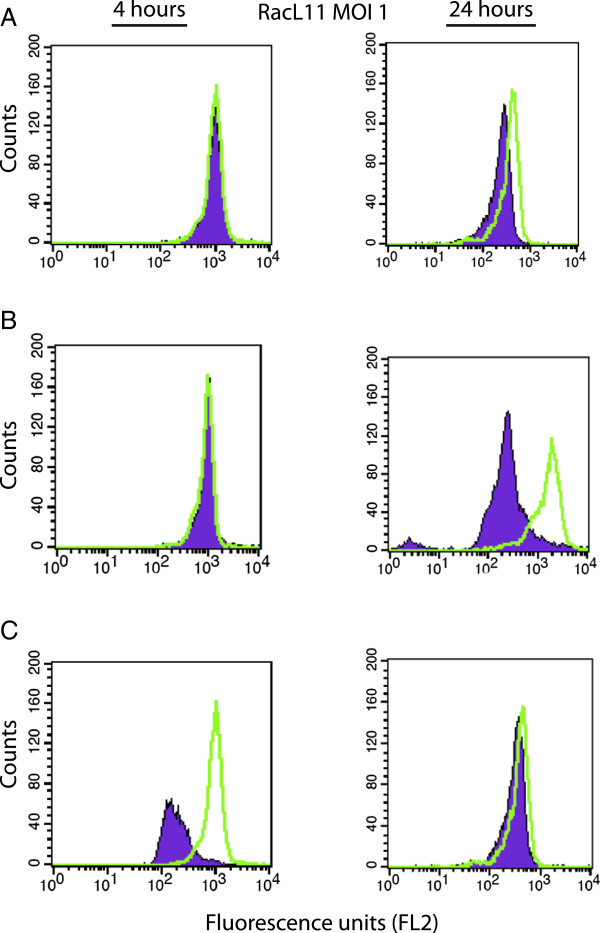
**Changes in mitochondrial membrane potential (ΔΨm) after EHV-1 infection.** Monocyte-enriched fractions of equine PBMC (5 × 10^5 ^cells) were infected for 4 (left panel) or 24 h (right panel) with the RacL11 strain of EHV-1 at an MOI of 1 (**A**) or 5 (**B**). Positive apoptosis and stimulation controls were sodium azide (25 mM) and LPS (10 ng/mL), respectively (**C**, only one time point for each control shown), whereas mock-infected cells served as a negative control (green overlay). Changes in ΔΨm were evaluated with DePsipherTM and flow cytometry. With this technique, decreased red fluorescence (FL2) indicates altered ΔΨm, an early marker of apoptosis. Data shown is representative of 3 different experiments. Marked changes in ΔΨm, indicative of apoptosis, were evident in cells infected with RacL11 at an MOI of 5 after 24 h and sodium azide controls, but not in cells infected with RacL11 for 4 h at either MOI or 24 h after EHV-1 infection with an MOI of 1 or treatment of cells with LPS.

To determine if the induced monocyte procoagulant activity was due to a secondary effect of virus-induced cell death, as reported for LPS-stimulated human monocytes [45], flow cytometric measurement of mitochondrial transmembrane potential (ΔΨm) was performed in EHV-1-infected monocyte-enriched fractions at 4 and 24 h after infection. Disruption of ΔΨm is an early and necessary event of apoptosis [38,39]. ΔΨm in gated monocytes did not change after 4 h of infection with all virus strains, regardless of the MOI used (Figure 4A and B left panel, only RacL11 shown), or with LPS stimulation (data not shown), in spite of pronounced increases in procoagulant activity at this time point (Figure 2A). At 24 h after infection, mild changes in ΔΨm were seen with all EHV-1 strains at an MOI of 1 (Figure 4A right panel, only RacL11 shown) but changes were minimal after LPS stimulation (Figure 4C, right panel). Marked changes in ΔΨm were seen 24 h after infection with all virus strains at an MOI of 5 (Figure 4B right panel, only RacL11 shown). Marked changes in ΔΨm were also seen at both time points with the sodium azide positive control (Figure 4C left panel, only 4 h shown).

**Figure 5:**
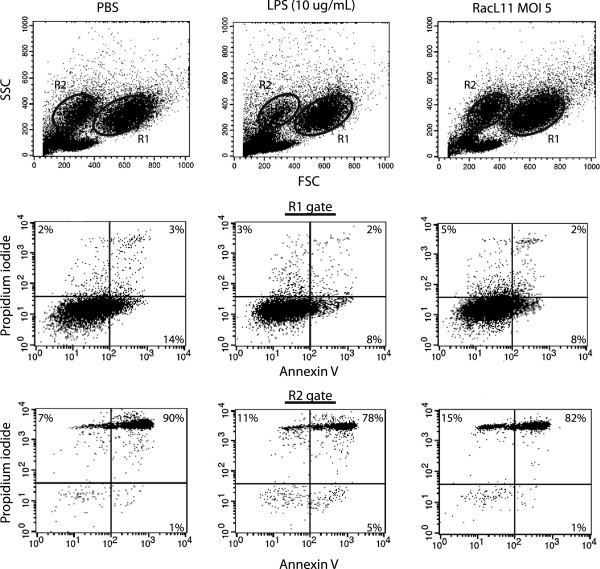
**Live-dead assay on EHV-1-infected or control-treated monocyte-enriched fractions of peripheral blood. **Representative flow cytometric analysis of Annexin V and propidium iodide staining of monocyte-enriched fractions of equine PBMC (5 × 10^5 ^cells) infected for 24 h with RacL11 (MOI of 5, right panel). Vehicle (PBS, left panel) and LPS (10 μg/mL, middle panel) served as negative and positive stimulation controls, respectively. Monocytes were identified and gated (R1) by their characteristic FSC and SSC. A second population of cells with lower FSC and similar SSC was identified and similarly gated (R2; upper panel). The percentage of dead cells in each gate was determined by combining the total percentage of cells positive for Annexin V (lower right quadrant), propidium iodide (upper left quadrant) or both Annexin V and propidium iodide (upper right quadrant) in fluorescence dotplots (lower two panels). In this representative experiment (of *n *= 4), the degree of cell death in the monocyte gate was similar for EHV-1-infected (15%), PBS- (19%) or LPS-treated (13%) cells. The R2 gate consisted mostly of dead cells (EHV-1: 98%; PBS: 98%, LPS: 94%).

Changes in ΔΨm are potentially reversible [39], thus we determined the percentage of dead cells (based on Annexin V staining and/or propidium iodide uptake). Monocytes were gated on their characteristic FSC and SSC (R1) after infection with RacL11 at an MOI of 1 or 5 for 4 or 24 h or treatment with vehicle (PBS), LPS (10 μg/mL) or sodium azide (Figure 5, Table 5). We found that a similar percentage of monocytes were reactive with Annexin V or exhibited propidium iodide uptake after EHV-1 infection at both MOI or after treatment with PBS or LPS at both time points (Figure 5, Table 5, only 24 h shown). A significantly higher proportion of gated monocytes were dead at 24 versus 4 h (19.3 ± 7.6% versus 9.5 ± 5.8%, *p* = 0.003, data of all treatments combined for each time point). Also, the total percentage of events within the monocyte gate (R1) was not significantly different between treatments at either time point. In contrast, there were fewer total events and more dead cells in the monocyte gate after sodium azide treatment at both time points (Table 5, only 24 h time point shown). We noticed that, after in vitro culture, there was an additional event population with similar SSC but lower FSC compared to monocytes (Figure 5, R2) that was absent in monocyte-enriched fractions evaluated immediately after purification (data not shown). A similar population of cells has been observed in LPS and calcium ionophore-stimulated human monocytes and corresponds to dead cells on live-dead staining [46]. We found that this additional population was, indeed, comprised of dead cells, regardless of infection or treatment (Figure 5, Table 5). Furthermore, the percentage of events within this gate was similar with all treatments at both time points, except for sodium azide, which had, on average, double the total events (Table 5). This increase in total events in the “dead cell” or R2 gate with sodium azide corresponded to the reduction of total events in the monocyte or R1 gate, implying the increased total R2 events after sodium azide treatment represented dead monocytes (originally in the R1 gate). We also tested if procoagulant activity was induced in cells treated with sodium azide and found no significant increase in procoagulant activity (data not shown). We concluded from the results that the increase in procoagulant activity in EHV-1-infected monocytes at 24 h after infection is unlikely due to overt apoptotic cell death.

**Table 5:** In vitro culture of monocytes induces cell death, which is not increased by EHV-1 infection

**Treatment**	**Monocyte gate**		**Dead cell gate**	
	**% total**	**% dead**	**% total**	**% dead**
Vehicle	54 ± 21	16 ± 3	9 ± 7	65 ± 29
LPS	55 ± 25	18 ± 15	6 ± 4	80 ± 18
RacL11 MOI 1	59 ± 17	14 ± 8	9 ± 7	79 ± 24
RacL11 MOI 5	59 ± 14	16 ± 10	7 ± 5	83 ± 17
Sodium azide	33 ± 26	31 ± 26	18 ± 19	92 ± 10

Live-dead assays with Annexin V and propidium iodide were performed on suspended monocyte-enriched fractions 24 h after EHV-1 infection with RacL11 (MOI of 1 and 5) or treatment with LPS (10 μg/mL) or vehicle (PBS). Sodium azide (25 mM) was used as a positive control for induction of cell death. The mean ± SD percentage of total cells and dead cells (expressing Annexin V or propidium iodide) was calculated in the monocyte and “dead cell” gate (R1 and R2 in Figure 5, respectively) (*n *= 4). Mean percentages for infected or treated cells were not significantly different from vehicle-treated controls (*p *> 0.05).

## Discussion

For the first time, this study shows that EHV-1 infection of equine monocytes induces procoagulant activity that is mediated by increased TF gene expression and availability. The data also suggests that virus-induced procoagulant activity is a two-step process: initial virus binding or uptake induces most of the activity, but virus replication is required for maximal activity. There is also a dose-related response, with higher infectious doses yielding higher procoagulant activity.

The increased monocyte procoagulant activity after virus infection was temporally and quantitatively associated with TF mRNA expression and dependent on addition of exogenous FVIIa. Similar findings were seen with LPS stimulation. This indicates that TF is the main driver of the observed activity and activity is mostly mediated through TF gene transcription with subsequent protein expression on monocyte surfaces, as reported for LPS stimulation [47]. The signaling events by which EHV-1 stimulates TF mRNA production are currently unknown, but the virus could trigger the same pathways that result in TF gene expression after LPS stimulation, including activation of MAP kinases and the transcription factors, NFκβ, activating protein-1 or early growth response protein-1, all of which were shown to bind to responsive elements in the TF gene promoter and activate transcription [48,49]. Further studies, however, are necessary to uncover the signaling pathways by which TF mRNA is upregulated after EHV-1 infection. At higher infectious doses of EHV-1, procoagulant activity was observed in monocytes within 1 h of infection and was even higher than that seen with LPS. This rapid increase in activity cannot entirely be attributed to de novo TF protein synthesis and it is possible that EHV-1 induces release of intracellular stores of TF or decrypts surface-expressed TF [50,51]. The majority of cellular TF is stored intracellularly within endosomes and Golgi vesicles, but studies in human monocytes and fibroblasts have shown that these stores can be mobilized rapidly with appropriate stimulation, including LPS [41,50].

We found that higher infectious doses of EHV-1 resulted in permeable mitochondrial membranes in equine monocytes 24 h after infection, but this was not concurrently associated with overt cell death. There are several potential explanations for these findings. It is possible that the cell death sequence is initiated at 24 h and would only fully manifest at a later time. In support of this explanation, we have observed that many monocytes are lysed 48 h after EHV-1 infection (MOI of 5; data not shown). Mitochondrial membrane changes are potentially reversible [39], but this has not been reported following virus infection. Herpesviruses can upregulate anti-apoptotic proteins, which could inhibit or delay cell death despite mitochondrial disruption [52]. Since the degree of overt cell death was similar between EHV-1-infected, mock-infected or LPS-stimulated equine monocytes and procoagulant activity was not increased in sodium azide-treated monocytes despite substantial cell death, we could not attribute the increased procoagulant activity at 24 h after EHV-1 infection to cell death. Our results also indicate that cultured equine monocytes undergo spontaneous cell death, which increases from 4 to 24 h of culture, but was not enhanced by virus infection or LPS stimulation (10 ng/mL or 10 μg/mL). Such spontaneous cell death has also been observed in human monocytes cultured in vitro [53,54]. Our results with LPS-treated equine monocytes differ from that previously reported with human monocytes, in which a lower dose of LPS (1 μg/mL) induced more cell death than was observed in untreated controls [45]. This could be due to species differences in the response to LPS. The procoagulant response after LPS stimulation was also highest at 24 h versus 4–6 h after treatment, as reported previously for horse monocytes [36]. The latter conflicting results may be explained by different experimental methods, since a two-stage amidolytic assay was used to measure procoagulant activity on the surface of living cells in this study whereas a clotting assay was used to measure procoagulant activity in whole cell lysates of equine monocytes in the previous study [36].

The data also revealed strain-dependent differences in generation of procoagulant activity in equine monocytes, with RacL11 consistently eliciting a stronger response than Ab4 or NY03. These strain-dependent differences were not attributable to the D_752_ polymorphism in the DNA polymerase. This polymorphism has been strongly associated with neuropathogenicity [25,27,32,33], possibly due to a leukocyte-associated viremia of higher magnitude and longer duration [25,27] or more efficient infection of CD172-positive mononuclear cells after invasion through the respiratory mucosa [2,29,44]. Interestingly, the D_752_ polymorphism in the virus DNA polymerase did affect the maximal procoagulant response induced by RacL11 but not by Ab4. The reason for this is unclear but it is unlikely to be due to virus yields or differences in replication, since previous studies have shown that strains harboring the D_752_ or N_752_ polymorphism yield similar amounts of virus [25,26]. The result does, however, support our findings with UV-inactivated or aphidicolin-treated virus showing that virus replication contributes to procoagulant activity with EHV-1. The reason for the higher procoagulant activity with RacL11 is unknown, but similar results have been reported for chemokine gene expression patterns, with RacL11 causing higher levels of CCL2 and CCL3 mRNA in equine PBMC than NY03 or Ab4 after 24 h of infection [55]. Since the observed differences in infection efficiency between RacL11 and Ab4 were small in this study, other factors are likely causative, such as variations in gene products between the virus strains used. The rapid induction of procoagulant activity and TF mRNA transcription suggests that early events in virus infection, such as attachment, entry, intracellular trafficking or transcription of immediate-early genes [56], may be mediating the observed procoagulant responses. RacL11 differs from the other two strains by partial or complete deletions in open reading frames 1 and 2, that encode early gene products, and also has a large deletion in the early gene product, ICPO, a powerful transcriptional transactivator [57]. To the authors’ knowledge, there are no EHV-1 strain differences in the gC envelope glycoprotein, which facilitates herpes simplex virus-induced procoagulant responses in human umbilical vein endothelial cells [58]. Future studies are required to elucidate which virus gene(s) are responsible for strain-dependent differences in procoagulant activity in equine monocytes.

Procoagulant activity was induced after addition of EHV-1 or EHV-4 to monocyte-enriched fractions. The effect was partly replication-dependent in the case of EHV-1, but not with EHV-4, as shown by inhibition with UV light inactivation of the virus and aphidicolin treatment of infected cells. EHV-4 does not appear to efficiently replicate in monocytes [43,44], which may explain the lack of replication-dependent procoagulant activity with this virus. However, since procoagulant activity independent of virus replication was seen with both viruses, early events such as virus attachment, entry or intracellular trafficking, appear sufficient to induce this response and may even be required for the maximal procoagulant activity that is induced by replication with EHV-1. Similar results have been reported with endothelial cell infection by herpes simplex virus and avian hemangioma retrovirus [59,60]. It is possible that the procoagulant response in monocytes is part of an innate immune response of host cells to virus infection. There is a mounting body of evidence that coagulation factors, including TF, can stimulate inflammatory and immune responses, through cleavage of protease-activated receptors (PARs) [61,62]. Interactions of EHV-1 glycoproteins with cell surface receptors or recognition of CpG-rich virus DNA by intracellular Toll-like receptors or cytosolic DNA-dependent RNA polymerase following virus binding and uptake may activate signaling pathways resulting in TF gene transcription and other immune responses, such as chemokine secretion [63,64]. The virus could also co-opt both innate immune receptors and the hemostatic system to support its own replication. For instance, both TF and thrombin, through activation of PAR2 and PAR1 respectively, increase the susceptibility of human umbilical vein endothelial cells to herpes simplex virus infection [65,66].

There is some controversy over which mononuclear cell in peripheral blood is responsible for the PBMC-associated viremia seen after EHV-1 infection. The cytotoxic T lymphocyte has been implicated as the main cell responsible for both EHV-1 latency and viremia after in vivo infection [5,67]. However, this study and others have shown that the virus is predominantly found in monocytes and B lymphocytes after in vitro infection of PBMC [25,26]. Furthermore, in vivo and in vitro studies with equine nasal explants suggest that the main targets of infection after uptake and replication within the nasal mucosa are CD172-positive mononuclear cells, which are either monocytes, dendritic cells or neutrophils [2,29,44]. It is possible that monocytes are the vehicle of virus transfer to endothelial cells in the placenta and central nervous system [3,6,7]. In support of this, EHV-1 virus gene products have been amplified from monocyte-enriched fractions of peripheral blood after in vivo infection [5].

Since TF triggers the initiation phase of coagulation in vivo, its induced expression on monocytes could contribute to the pathologic thrombosis associated with EHV-1 infection. However, systemic vascular thrombosis is not a typical finding in EHV-1-infected horses; rather, thrombosis has been observed in specific vascular beds, including arterioles of the nasal mucosa (after aerosol challenge), arterioles, venules and capillaries in the spinal cord and brain, and arterioles in the placenta [8-10]. These observations indicate that monocyte-associated TF is unlikely to be the sole cause of thrombosis in EHV-1-infected horses and other factors are involved. These factors could include, but are not limited to, upregulation of vascular bed-specific adhesion molecules, which mediate adhesion of TF-bearing monocytes to regional endothelial cells [6,7], high regional expression of procoagulant factors in infected monocytes and endothelial cells [16,17], and downregulated local anticoagulant defenses [16].

Collectively, our results show that various strains of EHV-1 induce procoagulant activity in equine monocytes as early as 1 h after infection. The procoagulant activity could contribute to the thrombosis observed in EHV-1-associated disease syndromes in horses, but could also be a mechanism by which the virus co-opts host responses to facilitate replication and dissemination in the animal. Since procoagulant activity does not require virus replication and also occurs with other herpesviruses that are not associated with thrombosis in vivo, it is also possible that the induced procoagulant activity in this inflammatory cell may be a natural component of the host defense against the virus. Elucidating the virus proteins and specific signaling pathways through which EHV-1 induces procoagulant responses in monocytes could provide valuable insight into general mechanisms of how viruses affect the innate immune response and co-opt the hemostatic system of an infected host.

## Abbreviations

ΔΨm: Mitochondrial transmembrane potential; cDNA: Complementary DNA; EHV-1: Equine herpesvirus type 1; EHV-4: Equine herpesvirus type 4; FVIIa: Activated factor VII; FSC: Forward scatter; FX: Factor X; FXa: Activated factor X; GFP: Green fluorescent protein; LPS: Lipopolysaccharide; MOI: Multiplicity of infection; PAR: Protease-activated receptor; PBMC: Peripheral blood mononuclear cells; PBS: Phosphate-buffered saline; SSC: Side scatter; TF: Tissue factor; UV: Ultraviolet.

## Competing interests

The authors declare that they have no competing interests.

## Authors’ contributions

WMY performed all experiments in the study, including virus and monocyte purification, monocyte infection, procoagulant activity assays, mRNA isolation, flow cytometric analysis, real time PCR and statistical analysis, and drafted the manuscript. KO participated in the design of the study, provided reagents and helped draft the manuscript. TS conceived of the study, participated in its design and co-ordination, helped with statistical analysis, performed the differential cell counts on cytospin smears of monocyte-enriched fractions, analyzed live-dead data, generated the figures, and helped draft the manuscript. All authors have read and approved of the manuscript.

## Authors’ information

Dr Yeo is a post-doctoral associate in Dr Stokol’s laboratory. Dr Osterrieder is the current director of the Institut für Virologie, Freie Universität Berlin. Dr Stokol is a board-certified clinical pathologist and Associate Professor at Cornell University.
